# Primary Care Strategies for Managing Musculoskeletal Pain: A Narrative Overview

**DOI:** 10.7759/cureus.88447

**Published:** 2025-07-21

**Authors:** Anusha Khan, Syed Osama Husain, Raminder Kaur, Ahmad Hashmat, Abid Nawaz Khan Adil, Alena Berkhamova, Saira K Awan, Giustino Varrassi

**Affiliations:** 1 Medical Ethics, Long Island Jewish Medical Center, Northwell Health, New York, USA; 2 General Medicine, North Manchester General Hospital, Manchester, GBR; 3 General Medicine, Manchester University National Health Service Foundation Trust, Manchester, GBR; 4 Family Medicine, The Wright Center for Graduate Medical Education, Scranton, USA; 5 Nephrology, Richmond University Medical Center, New York, USA; 6 Internal Medicine, California Health Science University, Clovis, USA; 7 Internal Medicine, Mercy Health, Toledo, USA; 8 Internal Medicine, Community Regional Medical Center, Fresno, USA; 9 Neurology, Advanced Neurology Professional Corporation, Brooklyn, USA; 10 Medicine, Kabir Medical College, Peshawar, PAK; 11 Pain Medicine, Fondazione Paolo Procacci, Rome, ITA

**Keywords:** chronic pain management, multidisciplinary approach, musculoskeletal pain, non-pharmacologic therapy, primary care, primary care practitioner

## Abstract

Musculoskeletal pain (MSP), including conditions such as osteoarthritis, low back pain, tendinopathies, and soft tissue disorders, is one of the most frequent reasons for primary care (PC) office visits and a leading cause of disability worldwide. MSP imposes a sizeable socioeconomic burden due to work absenteeism, healthcare utilization, and loss of productivity. Primary care practitioners (PCPs) are central to identifying and managing MSP, particularly in the early stages, where timely intervention can prevent chronicity and reduce disability. This narrative review presents clinical guidance and current evidence on the assessment and management of MSP in PC settings. Our study highlights the importance of a comprehensive, multidisciplinary approach that includes clinical evaluation with red and yellow flag screening and judicious use of imaging, followed by the selection of appropriate pharmacologic therapies such as nonsteroidal anti-inflammatory drugs (NSAIDs) and adjuvants based on pain severity and comorbidities. Non-pharmacologic interventions, such as psychological support, manual therapy, exercise therapy, and patient education, are central to long-term management. The review further emphasizes the integration of digital health tools and care models, such as the Subgroups for Targeted Treatment (STarT) Back approach, in improving care coordination and accessibility. Despite well-established guidelines, gaps in practice remain, including poor access to physiotherapy, limited consultation time, and the overuse of diagnostic imaging and opioids. Filling these gaps requires better clinician education, enhanced access to allied health services, and system-level support for providing conservative, patient-centered care.

## Introduction and background

Musculoskeletal pain (MSP) affects a wide demographic and includes conditions such as low back pain, osteoarthritis, tendinopathies, and soft tissue disorders. It contributes substantially to disability, work absenteeism, and healthcare burden globally. Primary care practitioners (PCPs) are central to identifying and managing MSP, particularly in the early stages, where timely intervention can prevent chronicity and reduce disability [1-3].

MSP is a prevalent clinical complaint that significantly affects individuals and contributes to a substantial burden [4,5]. Low back pain alone is currently the leading cause of years lived with disability (YLD) worldwide, with musculoskeletal disorders collectively accounting for a significant proportion of general practice consultations in high-income countries, including the UK, where they represent over 20% of primary care (PC) visits annually [6,7].

The socioeconomic impact of MSP extends beyond healthcare utilization. It is a major contributor to decreased productivity and long-term disability claims. The burden increases with age, obesity, and sedentary lifestyles. Occupational strain, particularly in jobs involving prolonged static postures or repetitive movements, could further increase the burden [8,9]. With the aging global population and rising levels of sedentary lifestyle, the incidence and prevalence of MSP are expected to continue increasing. Thereby, highlighting the need for effective PC strategies aimed at early identification, risk stratification, and comprehensive management [10].

The gatekeeping function of PC is critical in reducing unnecessary referrals, investigations, and specialist interventions. However, MSP management in PC is often challenged by limited consultation time, patient expectations for diagnostic imaging or quick treatment fixes, and variability in access to allied health services such as physiotherapy or psychological support [11-13]. These challenges highlight the need for clear, practical, and multidisciplinary management pathways tailored to PC.

Guidelines emphasize the importance of a biopsychosocial approach in the assessment and treatment of MSP. This approach incorporates biological, psychological, and social dimensions that influence pain perception and recovery [14]. For instance, "red flags" (i.e., fever, unintentional weight loss, neurological deficits) are used to screen for serious pathology, whereas "yellow flags" (i.e., anxiety, fear avoidance, poor coping) help identify patients at risk of poor outcomes due to psychosocial distress [15]. This holistic framework supports the integration of interventions such as psychological support, manual therapy, exercise therapy, and patient education, alongside judicious pharmacologic treatment, forming the foundation of high-value care [16,17].

Despite a robust evidence base that backs conservative, patient-centered approaches, there remains a gap between guideline recommendations and real world practice. Overuse of imaging, underutilization of physical therapy, and reliance on medications such as opioids continue in many PC settings, thereby raising concerns about safety, cost effectiveness, and long-term outcomes [18,19]. Emerging models of care, including stratified and multidisciplinary approaches, such as the Subgroups for Targeted Treatment (STarT) Back tool, offer promising frameworks to improve care quality and reduce disability [20]. Furthermore, the increasing use of digital health tools, such as virtual consultation platforms and telemedicine, offers new avenues for accessible patient care delivery, particularly in the underserved populations [21].

This narrative review aims to synthesize the clinical recommendations and current evidence for the assessment and management of MSP in PC. It presents key epidemiological trends, assessment principles, pharmacological and non-pharmacological treatment modalities, emerging models, and challenges in PC.

## Review

Methodology

This narrative review was conducted to synthesize clinical guidance and the current evidence on the assessment and management of MSP in PC settings. A structured approach was used to identify clinical guidelines, relevant literature, and expert consensus documents.

Search Strategy

A literature search for this narrative review was carried out using electronic databases including Google Scholar, MEDLINE, PubMed, and EMBASE. The search was limited to publications from 2010 to 2025. The key search terms included “primary care”, “musculoskeletal pain”, “low back pain”, “musculoskeletal disorders”, “management”, “NSAIDs”, “psychological interventions” and “exercise therapy”. Major guidelines were also hand searched from agencies such as the American Academy of Family Physicians (AAFP), Centers for Disease Control and Prevention (CDC), and World Health Organization (WHO) to identify additional relevant sources.

Inclusion Criteria

Reviews, articles, and guidelines addressing assessment, pharmacologic and non-pharmacologic management, and care models in PC that focused on adults with non-malignant musculoskeletal conditions and published in English were included.

Exclusion Criteria

Non-peer-reviewed articles, opinion pieces, and studies exclusively involving surgical interventions or specialist-only care were not included. Non-English language publications were also excluded.

Data Extraction and Synthesis

The review identified several key themes essential to understanding and improving the management of MSP in PC. These include the epidemiology and overall burden of MSP, highlighting the widespread impact on both healthcare systems and individuals. The principles of assessment in PC emphasize early and accurate diagnosis to guide effective management strategies. Several pharmacologic treatments were identified, with recommendations stratified by pain type and severity, to ensure tailored interventions. Additionally, this review examines evidence-based non-pharmacologic therapies. These include physical therapy and psychological support, which complement or are an alternative to medication in various ways. The importance of multidisciplinary care and the growing role of digital health innovations are also discussed as approaches to enhance patient outcomes. The review also addresses persistent challenges and barriers that hinder effective MSP management in PC, such as inadequate clinician training, time constraints, and resource limitation.

Evidence was synthesized qualitatively. Emphasis was placed on clinical relevance to PC practice, guideline-based recommendations, and consistency of findings across sources. Due to their methodological rigor, systematic reviews, clinical guidelines, and large scale epidemiological studies were given priority. This was done using strategies developed through consulting team members and published literature on the subject of biomedical reviews using the Scale for the Assessment of Narrative Review Articles (SANRA) scale [22]. The synthesized findings were then presented narratively.

Epidemiology and burden

MSP is globally one of the top contributors to YLD, with low back pain ranked as the leading cause [6]. In the UK, regional musculoskeletal disorders account for more than 20% of general practice consultations annually [13]. The disease burden increases with occupational strain, age, and sedentary lifestyle. Efficient PC management is essential in reducing unnecessary specialist referrals, healthcare burden, and imaging.

Assessment in primary care (PC)

Clinical Evaluation

A comprehensive history should be focused on pain onset, character, and functional impact. Red flags (i.e., fever, unintentional weight loss, trauma, and neurological deficits) should be recognized to rule out serious pathology [11]. Psychosocial factors or “yellow flags” (i.e., anxiety, fear avoidance, and poor coping) are equally important as they can influence recovery tracks [15].

Investigations

Laboratory investigations and imaging should be limited to cases with high clinical suspicion of serious underlying pathology. Guidelines discourage routine imaging for non-specific low back or neck pain, since it rarely changes management and can lead to unnecessary and over medicalization [14].

Pharmacologic management

Medications should be chosen based on the nature and severity of the pain and the patient’s comorbidities.

The current evidence on the efficacy of paracetamol in managing chronic MSP is limited. However, it is recommended as a first-line therapy for patients with mild-to-moderate symptoms. Paracetamol carries the advantages of accessibility, beneficial safety profile, and prevalent clinical familiarity [23].

Nonsteroidal anti-inflammatory drugs (NSAIDs) are effective for short-term symptom relief but require careful consideration due to their side effect profile, particularly the gastrointestinal (GI) toxicity and cardiovascular risks. GI sparing strategies include co-prescribing proton pump inhibitors (PPIs) and lowest effective dose [1,14].

Furthermore, topical agents such as NSAID creams or capsaicin can also offer pain relief with fewer systemic side effects due to minimal systemic absorption. This also significantly reduces the risk of GI toxicity [14]. Topical pain reducing creams are generally safer in cases where long-term use is warranted.

Adjuvant medications, including antidepressants (e.g., amitriptyline, duloxetine) and anticonvulsants (e.g., gabapentin), are also helpful options in PC for MSP, especially where the physician is managing cases of neuropathic pain and fibromyalgia [10,23]. These are also an alternative in patients that cannot tolerate NSAIDs. However, they must be prescribed with caution in older adult population. Regular monitoring for side effects is recommended to achieve maximum benefits while avoiding adverse effects [14].

Opioids are another option that need a lot of attention due to their drug abuse potential, dependence, and side effect profile. They should only be used for short durations, only in severe pain, and under strict monitoring [14]. A range of pharmacologic agents are available for managing chronic musculoskeletal pain, each with varying efficacy and risk profiles (Table 1).

**Table 1:** Pharmacologic management of musculoskeletal pain (MSP) NSAIDs: nonsteroidal anti-inflammatory drugs; GI: gastrointestinal Summary of commonly-used pharmacological treatments for chronic MSP. The table outlines key medication classes, typical use cases, potential benefits, and associated risks or considerations. Clinician should balance efficacy with safety, particularly for long-term management [1,10,14,23].

Medication class	Use case	Benefits	Risks/Considerations
Paracetamol	Mild pain	Widely available, safe at recommended doses	Limited evidence of efficacy for chronic MSP
NSAIDs (oral)	Short-term relief of moderate pain	Effective anti-inflammatory and analgesic effect	GI bleeding, cardiovascular risk; use lowest effective dose
Topical NSAIDs/Capsaicin	Localized pain, mild to moderate	Fewer systemic side effects	Skin irritation possible; limited penetration in deep tissues
Antidepressants	Neuropathic pain, fibromyalgia	E.g., amitriptyline, duloxetine help with pain modulation	Sedation, dry mouth, blood pressure changes (monitor closely)
Anticonvulsants	Neuropathic pain, fibromyalgia	E.g., gabapentin, pregabalin reduce nerve pain signals	Drowsiness, dizziness, weight gain
Opioids	Severe pain unresponsive to other treatments	Strong analgesia	Dependency risk, constipation, sedation; short-term use only

Non-pharmacologic management

Patient Education and Self-Management

The gist of PC is to both educate and empower the patients about their medical conditions. Patient education is essential in encouraging physical activity and reducing fear. Reassuring patients about the generally benign nature of MSP and promoting self-management can improve outcomes [10,24].

Exercise and Physical Therapy

Exercise is the cornerstone of treatment for most MSP conditions. Patient tailored programs, including aerobic activities, strength training, and flexibility have strong evidence for both improving function and reducing pain [10,14].

Manual Therapies

Manual therapy, including massage and spinal mobilization, may provide short-term symptom relief. These therapies should be made part of a broader active management strategy. Manual therapies, particularly osteopathic manipulative treatment, are both beneficial in relieving MSK pain and are generally considered safer options for pain management at the PC level [14,25,26]

Psychological Interventions

These include, but are not limited to, cognitive behavioral therapy (CBT), yoga, and meditation. CBT has shown benefits in managing chronic MSP and patients with high psychosocial distress [10,26,27]. Studies have shown varied evidence but patient engagement and willingness is of utmost importance for compliance to therapy [14].

Multidisciplinary and integrated care

Managing chronic MSP has shown promise when patients are offered or are engaged in integrated care models. This involves engaging occupational health professionals, physiotherapists, and psychologists. It has led to improved outcomes in chronic MSP. The stratified care model, such as the STarT for back pain allows for early risk stratification and targeted intervention, reducing healthcare costs and disability [11,20]. Furthermore, the increasing use of digital health tools and telemedicine offer new avenues for accessible patient care delivery, particularly in underserved populations [28].

Challenges

Although evidence-based guidelines are well-established, challenges in PC practice persist, such as inadequate access to physiotherapy, limited consultation time, and the excessive use of diagnostic imaging and opioids. Bridging these gaps calls for enhanced clinician training, improved availability of allied health services, and systemic support to promote conservative, patient-centered care [2,14].

## Conclusions

MSP is a prevalent clinical complaint that significantly affects individuals, contributing to a substantial personal and societal burden. PCPs, as a first point of contact, play a crucial role in the management of MSP. While medications such as NSAIDs, antidepressants, and topical agents can offer symptom relief, they must be prescribed judiciously. Furthermore, non-pharmacologic interventions such as manual therapies, exercise therapy, psychological support, and patient education constitute the cornerstone of effective long-term MSP management. Although evidence-based guidelines exist, practical challenges continue to limit their consistent application, including poor access to physiotherapy, limited consultation time, and the overuse of diagnostic imaging and opioids. Filling these gaps requires better clinician education, enhanced access to allied health services, and system-level support for providing conservative, patient-centered care.
